# A Low-Viscosity, Recyclable Polymer-Based Binder Strategy for Metal FDM: Toward High Powder Loading, Sustainable Processing, and Comprehensive Characterization of 17-4PH Stainless Steel Parts

**DOI:** 10.3390/polym17192575

**Published:** 2025-09-24

**Authors:** Sheyda Khazaee, Elie Bitar-Nehme, Rachid Boukhili, Jovan Kostenov, William Regnaud, Etienne Martin

**Affiliations:** 1 Mechanical Engineering Department, Polytechnique Montréal, 2500 Chemin de Polytechnique, Montréal, QC H3T 1J4, Canada; 2 Dyze Design, 1505 Saint-Thomas Suite 100, LeMoyne, QC J4P 3S2, Canada

**Keywords:** metal fused deposition modeling, paraffin wax binder, feedstock rheology, 17-4PH stainless steel, additive manufacturing

## Abstract

In metal fused deposition modeling (FDM), performance is governed by feedstock formulation, most critically the metal solid loading, while binder selection is constrained by environmental impacts and limited recyclability. This study investigates the development and performance of highly filled 17-4PH stainless steel (17-4PH) feedstocks formulated with a low-molecular-weight polymer binder system, specifically designed for FDM in metal additive manufacturing (AM). The binder system, composed of low-cost, recyclable paraffin wax and stearic acid, was used to prepare feedstocks containing 93.0–96.0 wt.% metal powder. Rheological analysis indicated that intermediate powder loadings (95.0–95.5 wt.%) yielded optimal shear-thinning behavior, essential for stable extrusion during printing. Printing trials identified 95.5 wt.% as the critical powder loading, delivering superior print fidelity and structural integrity relative to both under-filled (93.0–94.5 wt.%) and overfilled formulations. Green part characterization revealed increased density and flexural modulus with rising powder content, while thermal debinding and sintering trials indicated enhanced thermal stability and dimensional retention at higher loadings. The as-sintered specimens from the 95.5 wt.% feedstock achieved a relative density (RD) of 96.5% and significantly improved mechanical performance, including an ultimate tensile strength (UTS) of 758 MPa and 5.2% elongation, clearly outperforming the 95.0 wt.% variant. Tribocorrosion testing further validated these improvements, with the higher-density samples showing a lower coefficient of friction and a reduced wear coefficient of 2.1 × 10^−5^ mm^3^·(N·m)^−1^ in 3.5% NaCl solution.

## 1. Introduction

Metal additive manufacturing (AM) has emerged as a disruptive and transformative technology for fabricating complex, high-performance components. Its ability to enable design freedom, reduce material waste, and produce near-net-shape parts has driven widespread adoption across various industries, including aerospace, biomedical, and automotive sectors [1]. This technology enables the production of complex metal structures by utilizing a wide variety of alloy compositions, including nickel [2,3,4,5,6], steel [7,8,9,10,11], magnesium [12,13,14], aluminum [15,16,17,18,19,20] and titanium [21]. Among the diverse AM techniques, fused deposition modeling (FDM) adapted for metal feedstocks has gained considerable attention due to its simplicity, low cost, and accessibility, offering a viable pathway for manufacturing metal parts with intricate geometries [22,23,24].

In metal FDM, a thermoplastic feedstock containing a high-volume fraction of metal powder is extruded layer-by-layer to form a green part. Subsequent thermal debinding removes the polymer binder, and sintering at high temperatures densifies the structure into a fully metallic component [22,25].

A commonly used metal in FDM applications is 17-4PH stainless steel (17-4PH), known for its excellent mechanical strength, corrosion resistance, and suitability for structural and functional engineering applications [26,27,28]. In metal FDM, the binder system plays a critical role in determining the flow properties, printability, debinding behavior, and the environmental footprint of the feedstock [29]. Most existing studies focus on binder systems based on synthetic polymers such as polyethylene (PE), polypropylene (PP), and ethylene-vinyl acetate (EVA), which, despite their functional effectiveness, are non-biodegradable, potentially hazardous during thermal decomposition, and not environmentally sustainable [30,31]. An ideal binder system must exhibit low melting temperature, smooth melt flow, effective wetting of metal particles, easy removal during debinding, and minimal ecological impact [32]. Paraffin wax (PW), a recyclable and relatively low-toxicity material, satisfies many of these criteria. Compared to synthetic polymers, PW offers environmental benefits due to its recyclability, low toxicity, and potential for reuse in closed-loop systems [33,34,35]. However, to the best of the author’s knowledge, the application of PW-based binder systems in FDM of steel materials has received limited attention. The relatively high weight and mechanical demands of steel parts typically necessitate the use of stronger backbone binders. One effective strategy to address this challenge is to increase the volume fraction of metallic powder, thereby enhancing interparticle friction and improving the thermal stability and dimensional integrity of the printed part during debinding and sintering [36,37].

While metal FDM holds significant promise for fabricating high-performance components, its success largely depends on the careful formulation of the feedstock, particularly the optimization of metal powder content. Higher metal loading generally enhances mechanical strength, dimensional accuracy, densification, and reduces sintering shrinkage. However, this also increases the feedstock’s viscosity, negatively impacting flow behavior, extrudability, and overall printability. For example, Hasib et al. [38] showed that increasing Ni-Cu content reduced shrinkage by up to 76.0%, though extrusion failed beyond 63.4 vol% due to high viscosity. Oh et al. [39] similarly found that trimodal iron powders achieved optimal sintered quality at 70.0 vol.%, with further increases compromising flow and part integrity. Abel et al. [40] demonstrated successful fabrication of NiTi components at 63.0 vol% loading but highlighted issues with impurity uptake affecting functional performance.

To mitigate the challenges posed by high metal powder loading, the binder system plays a pivotal role in controlling feedstock rheology and ensuring printability. An optimized binder composition can significantly reduce viscosity and improve processability, even at elevated solid loadings. Cano et al. [41], for example, developed a binder system for zirconia FDM using acrylic acid-grafted high-density polyethylene (HDPE) blended with stearic acid (SA), achieving low viscosity, high tensile strength, and efficient debinding. Similarly, Momeni et al. [42] investigated binder systems based on linear low-density polyethylene (LLDPE) for Nd-Fe-B metal injection molding (MIM) feedstocks. They demonstrated that optimized binder formulation enabled stable flow behavior and reduced viscosity at high powder loadings, while also achieving enhanced magnetic and physical properties in the final parts. However, their work is limited to rare-earth magnetic powders and relies heavily on LLDPE as a polymeric backbone. Such backbones often require higher processing temperatures and contribute to higher carbon residues.

To the best of our knowledge, no prior study on 17-4PH has used PW and SA as the sole binder components in either MIM or extrusion-based AM. This study addresses that gap by formulating and evaluating feedstocks composed of PW (as the primary binder) and SA (as surfactant), targeting high metal powder loadings ranging from 93.0 wt.% to 96.0 wt.%. The goal is to determine the maximum viable metal content that maintains acceptable printability while improving the structural and functional properties of the final parts. To this end, the feedstocks are systematically characterized in terms of rheological behavior, print quality, and the structural integrity of printed components. Further assessments include the physical and mechanical properties of green and as-sintered parts, along with evaluations of wear and corrosion resistance and fractographic characteristics.

## 2. Experimental Procedures

### 2.1. Materials

**Figure 1:**
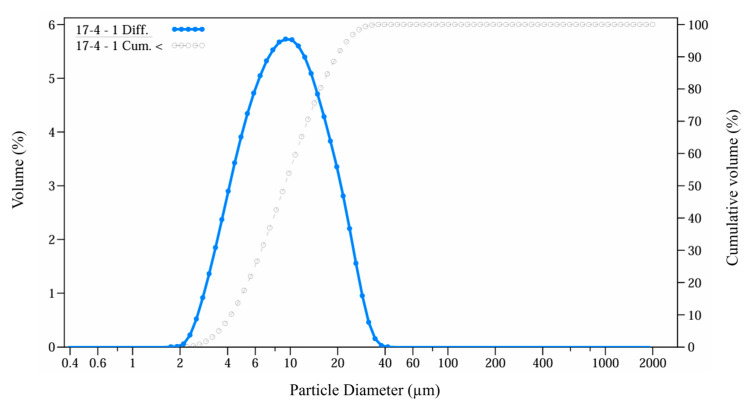
Size distribution of 17-4PH metal powder.

The feedstocks used in this study were prepared by combining gas-atomized 17-4PH metal powder with wax-based binders. The chemical composition of the metal powder is presented in Table 1. The 17-4PH powder exhibits a near-spherical morphology with smooth surfaces. The particle-size distribution, shown in Figure 1, spans 4–20 µm (*D*_10_–*D*_90_) with a median size *D*_50_ ≈ 9 µm. Particle-size distribution was measured by laser diffraction technique employing a Coulter LS 200 (Beckman Coulter, Krefeld, Germany) in accordance with ASTM B822-10, using wet dispersion in methanol and Fraunhofer optical model with run length of 60 s. The organic binder comprised PW (Sigma-Aldrich, 327204, CAS 8002-74-2, mp 53–58 °C, Oakville, ON, Canada) as the primary matrix to uniformly disperse the metal powder and preserve the shape of the printed component, while SA (Sigma-Aldrich, W303518, assay ≥ 95%, mp 67–72 °C, Oakville, ON, Canada) acted as a surfactant.

### 2.2. Feedstock Preparation

In this study, feedstocks were prepared with metal powder loadings ranging from 93.0 to 96.0 wt.%. The lower limit of 93.0 wt.% was chosen to prevent excessive binder content, as the low-viscosity PW and SA binder system can compromise the structural stability of printed parts at insufficient solid loading. Conversely, powder contents exceeding 96.0 wt.% were excluded due to poor binder wetting and dispersion, which hinder homogeneous feedstock formation. Throughout the formulations, the PW-to-SA weight ratio was maintained at approximately 5:2, a proportion previously shown to provide an optimal balance of flowability, particle adhesion, and effective debinding in metal FDM applications [43,44]. The specific compositions of the feedstocks used in this work are summarized in Table 2.

The feedstock preparation was carried out using a high-speed overhead mixer (Model RW 20 Digital, IKA Werke GmbH & Co. KG, Staufen, Germany) under controlled thermal conditions to ensure homogeneity and moisture elimination. First, the metal powder was accurately weighed and preheated at 100 °C for 30 min on a hot plate to remove any residual moisture. Subsequently, the pre-weighed SA was introduced and thoroughly mixed with the preheated metal powder to promote surface wetting and uniform distribution. Finally, PW was added to the mixture, and the blending process continued at 150 °C and 500 rpm for 1 h to achieve a homogeneous feedstock. Following mixing, the composite material was cooled to room temperature and mechanically ground to form granulated pellets suitable for feeding into the Metal FDM system.

### 2.3. Rheological Measurement

Rheological analysis was conducted to evaluate the flow behavior and processability of the feedstocks, which is critical for ensuring uniform dispersion, printability, and defect-free fabrication. The measurements were performed using a rotational rheometer (MCR501, Anton Paar GmbH, Graz, Austria) equipped with a 25 mm diameter parallel plate configuration and a fixed gap of 1 mm. Shear viscosity was assessed at a constant temperature of 90 °C over a shear rate range of 0.1 to 1000 s^−1^.

### 2.4. FDM Printing

The feedstocks were printed using the Pulsar™ precision pellet extruder (Dyze Design, Montreal, QC, Canada) which is capable of handling high-viscosity materials with consistent flow control. To identify the optimal printing conditions for achieving high dimensional accuracy and structural integrity, a Taguchi design of experiments (DOE) approach was implemented using Minitab^®^ Statistical Software (version 21.3, Minitab, LLC, State College, PA, USA). This method facilitated the systematic evaluation of key process parameters, including nozzle temperature, printing speed, and material flow rate. The experimental matrix for the selected parameters is presented in Table 3.

Each feedstock composition was printed according to the DOE scheme, allowing the determination of parameter sets that yielded the most accurate and mechanically stable parts. During all printing trials, the nozzle diameter and layer height were held constant at 1.2 mm and 1.0 mm, respectively, to ensure comparability across samples.

### 2.5. Post-Printing Thermal Processing

**Figure 2:**
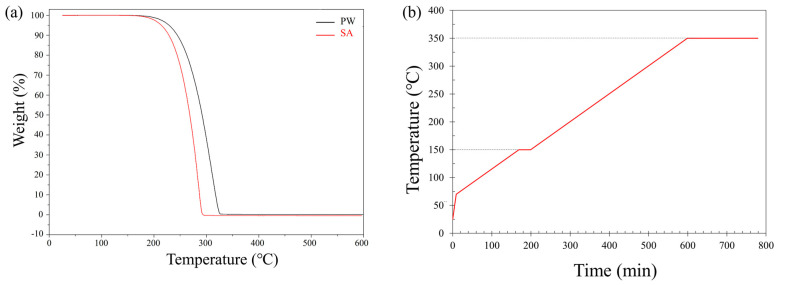
(**a**) TGA curve for binder components, (**b**) Debinding process for the printed parts under air atmosphere.

To eliminate the organic binder and obtain dense, fully metallic parts, the printed samples underwent a two-stage thermal processing comprising debinding followed by sintering. The debinding profile was designed based on thermogravimetric analysis (TGA) of the binder components, as illustrated in Figure 2a. TGA was performed using a TA Instruments Q500 (TA Instruments, New Castle, DE, USA) under nitrogen atmosphere (60 mL·min^−1^), with a sample mass of 15 mg, the temperature was ramped from room temperature to 600 °C at 10 °C·min^−1^ and subsequently cooled to room temperature at 10 °C·min^−1^ with no dwell time. The analysis showed that binder degradation begins at approximately 180 °C, with complete decomposition of PW occurring around 320 °C.

Debinding was conducted in a Heratherm oven (Thermo Fisher Scientific, Waltham, MA, USA) under atmospheric conditions, following the thermal profile shown in Figure 2b. The samples were first heated at 5 °C·min^−1^ to 70 °C, the melting point of the wax-based binder. To reduce thermal stress and maintain part geometry, the heating rate was then lowered to 0.5 °C·min^−1^. The temperature gradually increased to 150 °C and held for 30 min to promote early-stage degradation. Finally, the temperature was increased to 350 °C and maintained for 3 h to ensure complete removal of the binder without compromising structural integrity.

Following debinding, sintering was carried out to densify the printed parts and establish strong metallurgical bonds between metal particles. Based on information from relevant literature sources, a sintering temperature of 1340 °C was selected to ensure sufficient solid-state diffusion while avoiding the melting point [45].

**Figure 3:**
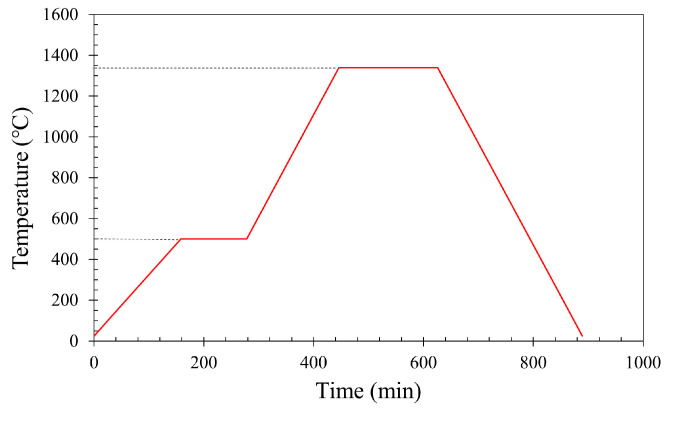
Sintering process for 17-4PH debinded print parts under hydrogen atmosphere.

The sintering process, illustrated in Figure 3, was conducted in a Carbolite tube furnace (Carbolite Gero Ltd., Hope, Derbyshire, UK) under a dry hydrogen (dew point ≤ −40 °C) to provide an actively reducing atmosphere for oxide removal on Cr-containing stainless steel powders and to promote densification [46]. The heating profile consisted of an initial ramp of 3 °C·min^−1^ to 500 °C, held for 2 h to remove any residual binder decomposition products. The temperature was then increased at 5 °C·min^−1^ to 1340 °C and maintained for 3 h to promote densification. Controlled cooling to room temperature followed to avoid thermal shock and preserve part integrity.

### 2.6. Scanning Electron Microscopy

Scanning Electron Microscopy (SEM) was used to examine (i) fracture surfaces of the tensile-tested, sintered specimens, (ii) powder-binder homogeneity in the mixed feedstocks, and (iii) the as-sintered microstructure. Fracture surfaces were observed as fractured (no additional preparation). For feedstock homogeneity, a small portion of the mixed feedstock was molded to a flat pellet and imaged on the molded surface. For microstructure observation, standard metallographic preparation procedure was followed, including grinding successively from #120 to #4000 grit papers, followed by polishing with 6 µm and 1 µm diamond suspensions, and then a final polish with colloidal silica (OP-S) to achieve a mirror finish.

All observations were performed on a JEOL JSM-7600F field-emission SEM (JEOL Ltd., Tokyo, Japan). under high vacuum. Backscattered electron (BSE) imaging was used for microstructure and phase contrast. Typical accelerating voltages were 5 kV and 15 kV. Scale bars were calibrated and are reported in figure captions.

### 2.7. Mechanical and Physical Analysis

Mechanical testing was performed at room temperature on an MTS Series 810 servohydraulic system (MTS Systems Corporation, Eden Prairie, MN, USA). Load cells of 25 kN and 100 kN were used for three-point bending of green parts and tensile testing of sintered parts, respectively. For each feedstock composition, five rectangular bars were prepared for flexure and three subsize dog-bones for tension to ensure consistency and reproducibility. Flexural testing followed ASTM D790 using bars of 127 × 12.7 × 3.2 mm^3^, the support span was set to *L* = 16 *d* (≈51.2 mm), and the crosshead speed was calculated from the standard equation, yielding approximately 1.4 mm·min^−1^ for *d* = 3.2 mm. Tensile testing followed ASTM E8/E8M on flat subsize dog-bone specimens with gauge length *L*_0_ = 25 mm, gauge width ≈ 6 mm, and thickness ≈ 3 mm after machining, strain was measured with a clip-on extensometer. The crosshead speed for yield determination was *v* ≈ 0.015 *L*_0_, which gives approximately 0.375 mm·min^−1^.

Bulk density was measured by the sealed-coating Archimedes method (ASTM B962). Each specimen was first weighed dry in air ($m_{d}$), then sealed with a thin paraffin layer and reweighed in air ($m_{2}$) and finally weighed while immersed in deionized water at 23 °C ($m_{3}$). Density was computed using Equation (1), relative density (RD) was obtained by normalizing to the theoretical density of 17-4PH, Equation (2).

(1) $$\rho_{bulk}=\frac{{\rho_{f\;}m}_{d}}{{(m}_{2}-m_{3})-(\frac{\rho_{f\;}}{\rho_{c\;}})(m_{2}-m_{d})}$$

(2) $$RD\;(\%)=100\times \frac{\rho_{bulk}}{\rho_{theoretical}}$$

The symbols used in Equations (1) and (2) are defined as follows. $m_{d}$ is the dry mass in air (g). $m_{2}$ is the mass in air after sealing with paraffin (g). $m_{3}$ is the immersed mass of the sealed specimen in deionized water (g). $\rho_{f\;}$ is the fluid density at 23 °C, with water ≈ 0.9975 g·cm^−3^. $\rho_{c\;}$ is the paraffin coating density at 23 °C. $\rho_{theoretical}$ is the theoretical density of 17-4PH, taken as 7.78 g·cm^−3^.

Results for mechanical and physical tests are reported as mean ± standard deviation, and error bars reflect one standard deviation.

### 2.8. Tribocorrosion

Wear tests under open circuit potential (OCP) conditions were performed using a custom-designed HTT-800 tribometer (Tricomat Inc., Quebec, QC, Canada). A three-electrode electrochemical configuration was employed, with the test specimen serving as the working electrode and a standard calomel electrode (SCE) as the reference. All tests were conducted at room temperature in a 3.5 wt.% NaCl aqueous solution, simulating a corrosive environment. A 4 mm diameter alumina ball was used as the counterbody and brought into contact with the sample surface under controlled conditions. Prior to initiating sliding, the electrochemical system was allowed to stabilize at its natural OCP for 3600 s, during which the potential evolution was monitored using a potentiostat. Reciprocating sliding motion was applied during the wear tests, with a stroke length of 5 mm, a frequency of 1 Hz, and a normal load of 2.25 N. Each test was run for 3600 s. Upon completion, samples remained immersed in the electrolyte for an additional 1800 s to allow for surface re-passivation. Post-test characterization of the wear scars, including surface morphology and wear volume, was conducted using a Bruker ContourX-100 optical profilometer (Bruker Corporation, Billerica, MA, USA).

## 3. Results and Discussion

### 3.1. Feedstock Characterization

**Figure 4:**
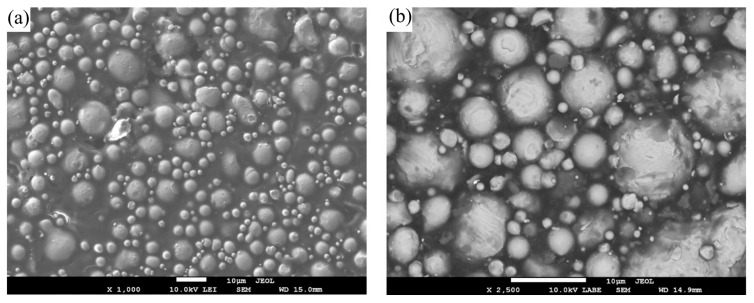
(**a**,**b**) SEM images of feedstock F2 (before printing) at different magnifications, showing uniform powder-binder dispersion and near-spherical 17-4PH powder morphology (smooth surfaces, occasional satellites) with continuous binder coverage of the particles.

As illustrated in Figure 4, the SEM micrographs of the F2 feedstock formulation, containing 94.0 wt.% metal powder, demonstrate a uniform distribution of metal particles and effective binder coverage. This microstructural homogeneity is indicative of successful mixing and is essential for consistent extrusion behavior and defect-free sintering. All feedstock formulations listed in Table 2 exhibited similar homogeneous microstructures, with F2 shown here as a representative example.

**Figure 5:**
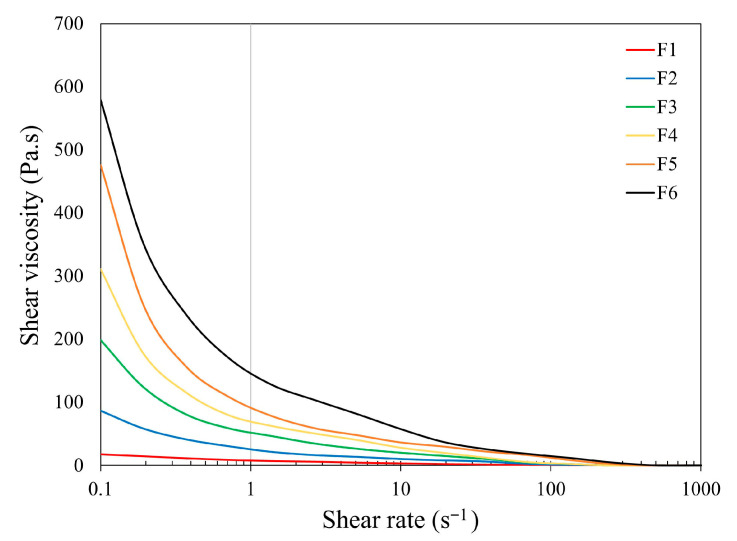
Viscosity diagram of the feedstocks at different shear rates at 90 °C.

The rheological properties of the prepared feedstocks were evaluated using shear viscosity measurements over a shear rate range of 0.1–1000 s^−1^ at 90 °C, as illustrated in Figure 5. This shear rate range covers the conditions typically encountered in the nozzle during FDM extrusion, while the test temperature approximates the actual extrusion temperature used during printing. All formulations exhibited clear shear-thinning behavior, characterized by a decrease in viscosity with increasing shear rate. This pseudoplastic flow behavior is typical of highly filled polymeric systems and is beneficial for extrusion-based AM processes, such as FDM, as it facilitates smoother flow through the nozzle while maintaining shape retention after deposition [47,48,49].

The viscosity increased significantly with rising metal powder content, ranging from 93.0 wt.% to 96.0 wt.%, due to the increased solid loading and intensified particle-particle interactions within the binder matrix. These observations align with previous studies, including the findings of Strano et al. [48], who reported a similar trend for metal-filled feedstocks. The elevated viscosity at 96.0 wt.% is attributed to the limited mobility of the binder phase, which can hinder material flow, increase the risk of clogging, and compromise printing stability [47].

Conversely, while lower metal contents (e.g., 93.0–94.5 wt.%) resulted in reduced viscosity and facilitated flow, these formulations exhibited poor printing stability at processing temperatures above the binder’s melting point. Phenomena such as stringing, inconsistent extrusion, and poor layer adhesion were observed, likely due to insufficient resistance to deformation and lack of structural support during printing [48,49,50].

Overall, a moderate viscosity range was found to be preferable for achieving stable, defect-free FDM processing. Feedstocks with intermediate metal contents (e.g., 95.0 wt.%, 95.5 wt.%) provided a balanced flow behavior, low enough viscosity to enable extrusion yet high enough to maintain dimensional accuracy and resist deformation during printing [51].

### 3.2. FDM Printability and Process Parameter Optimization

To evaluate the printability of each feedstock composition, nine predefined sets of printing parameters (listed in Table 3) were tested. For each feedstock, the parameter set that produced the most dimensionally accurate and structurally stable printed part was selected as optimal and is summarized in Table 4. Notably, feedstocks containing 93.0–94.5 wt.% metal powder exhibited slight structural instability, attributed to their relatively low viscosity from the binder system and reduced solid loading, even under optimized printing conditions. It is important to note that the aim of this study was not to establish a comprehensive processing window, but rather to facilitate a comparative assessment of different feedstock formulations. Therefore, the optimal parameters were chosen from within the predefined range, based solely on print quality, process stability, and dimensional precision consistent with the original computer-aided design (CAD) model.

As the metal content increased from 93.0 wt.% to 95.5 wt.%, feedstock viscosity also increased, necessitating adjustments in the printing parameters to maintain consistent extrusion and quality. Specifically, higher metal loadings required increased printing temperatures and flow rates to compensate for reduced flowability, while the printing speed was reduced to improve deposition control and reduce the risk of defects such as stringing or under-extrusion. The nozzle diameter (1.2 mm) and layer height (1 mm) were kept constant throughout the tests.

**Figure 6:**
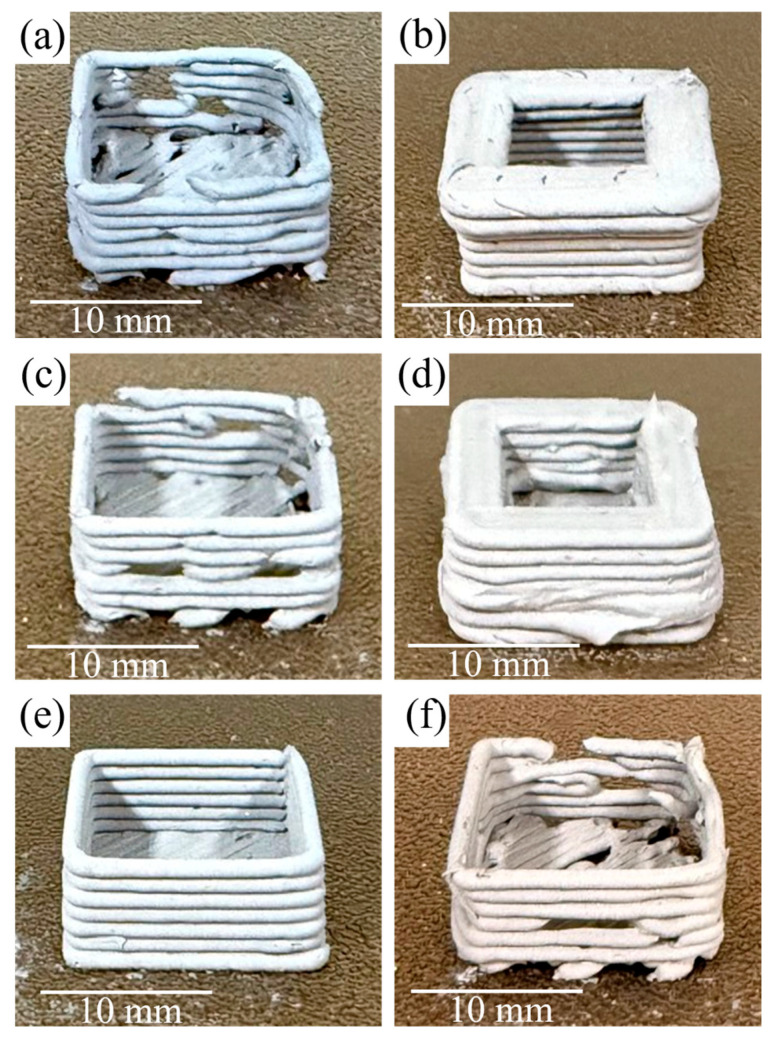
Effect of process parameters on green-part quality (**a**) low printing temperature: poor fusion and inconsistent extrusion, (**b**) high printing temperature: excessive flow and deformation, (**c**) low flow rate: under-extrusion and incomplete layers, (**d**) high flow rate: over-extrusion and surface irregularities, (**e**) low printing speed: stable, uniform deposition (time-consuming), (**f**) high printing speed: loss of fidelity and structural instability. Scale bars: 10 mm.

**Figure 7:**
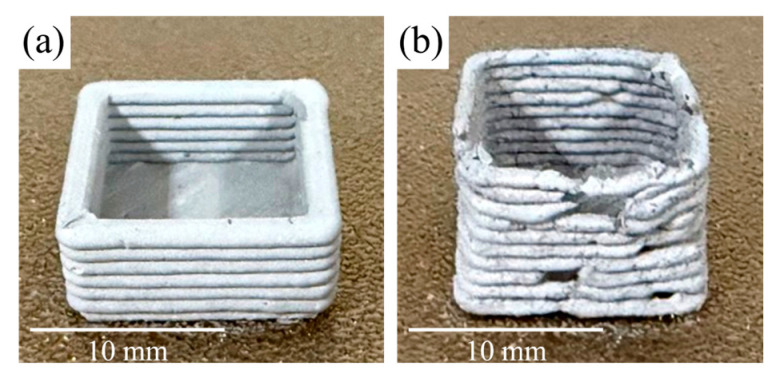
Images of green parts printed from (**a**) 95.5 wt.% metal feedstock (F5, optimized parameters) and (**b**) 96.0 wt.% metal feedstock (F6). Scale bars: 10 mm.

Figure 6 illustrates representative printing defects, such as inconsistent extrusion, sagging, or dimensional inaccuracy, using F5 feedstock, which were observed under non-optimal parameter sets, demonstrating the need for careful selection of conditions. Figure 7 shows the effect of metal content on print quality. The 95.5 wt.% metal sample (Figure 7a) printed with optimized parameters displays uniform layers and good structural integrity. In contrast, the 96.0 wt.% metal feedstock (Figure 7b) had excessively high viscosity and poor extrudability, resulting in unstable and discontinuous flow. Even at the maximum tested temperature of 170 °C, the feedstock could not be printed consistently, and the high temperature altered its appearance. Due to these issues, this composition was excluded from further processing.

### 3.3. Green Part Properties

**Figure 8:**
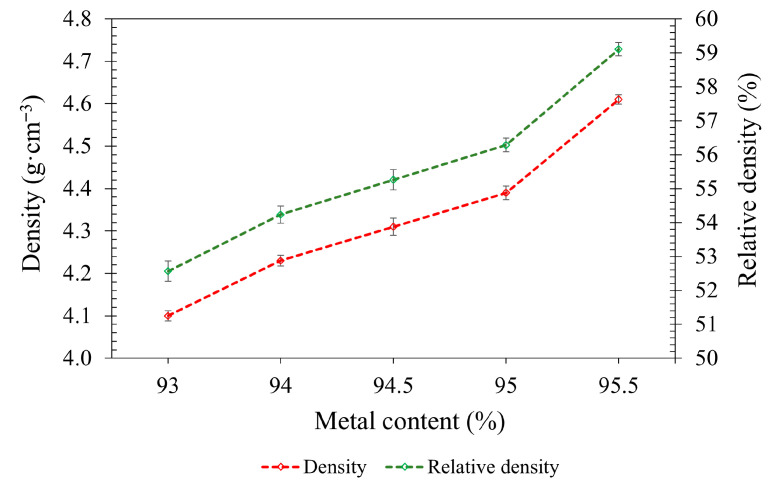
Density and RD values for the green printed parts.

Figure 8 shows that both the green density and RD increased with higher metal content, rising from 4.1 g·cm^−3^ and 52.5% at 93.0 wt.% to 4.6 g·cm^−3^ and 59.1% at 95.5 wt.%, respectively. This trend reflects the higher density of metal powder compared to the binder, leading to improved particle packing and reduced porosity. A particularly sharp increase was observed between 95.0 and 95.5 wt.%, where green density jumped from 4.4 to 4.6 g·cm^−3^. This suggests that the feedstock may be approaching the critical powder loading, where small increases in metal content can significantly enhance green part compaction. These findings are generally consistent with previous studies highlighting the influence of metal loading on green part density in highly filled feedstock systems [52,53].

**Figure 9:**
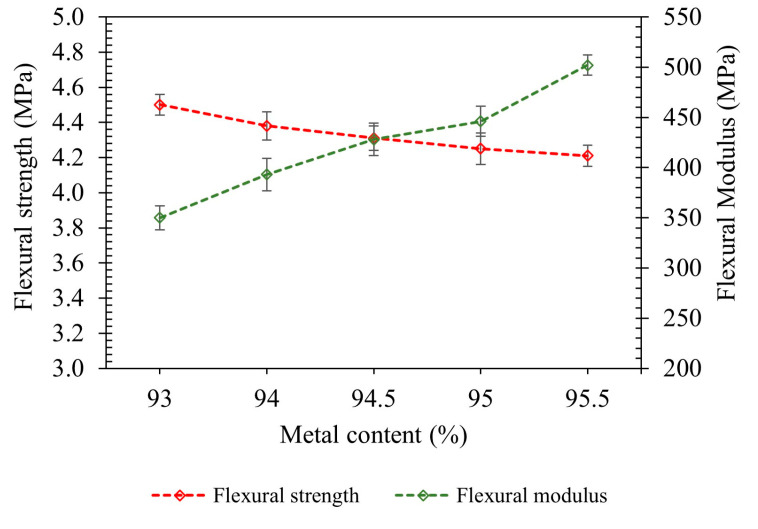
Flexural strength and flexural modulus of the green printed parts. Error bars indicate standard deviation based on five specimens per condition.

Figure 9 presents the flexural strength and modulus of green parts fabricated with varying metal content (93.0–95.5 wt.%) based on three-point bending tests. As metal content increased, the flexural modulus rose consistently, from 385 MPa at 93.0 wt.% to 512 MPa at 95.5 wt.%, indicating a steady increase in stiffness due to improved particle packing and the dominance of the rigid metal phase [54,55].

In contrast, flexural strength exhibited a slight decrease, from 4.5 MPa to 4.2 MPa across the same range. This moderate reduction can be attributed to limited binder continuity at higher powder loadings, which weakens interparticle bonding. Similar behavior has been reported by Bhavith et al. [56] in epoxy-cast iron composites, where increasing filler content altered the failure mode from ductile to brittle [57,58].

However, it is important to note that the decrease in strength is not substantial, and the green part with 95.5 wt.% metal content still maintains a flexural strength of 4.2 MPa, which is considered acceptable for safe handling and structural integrity during debinding. These results highlight the need to balance powder loading to achieve both high stiffness and adequate green strength for reliable processing in metal AM [59].

### 3.4. Debinding and Sintering Stability

The debinding process is critical in metal AM, as it removes the binder from green parts, leaving behind a fragile structure composed primarily of metal particles. The structural integrity of these parts during debinding is heavily influenced by the metal powder content.

**Figure 10:**
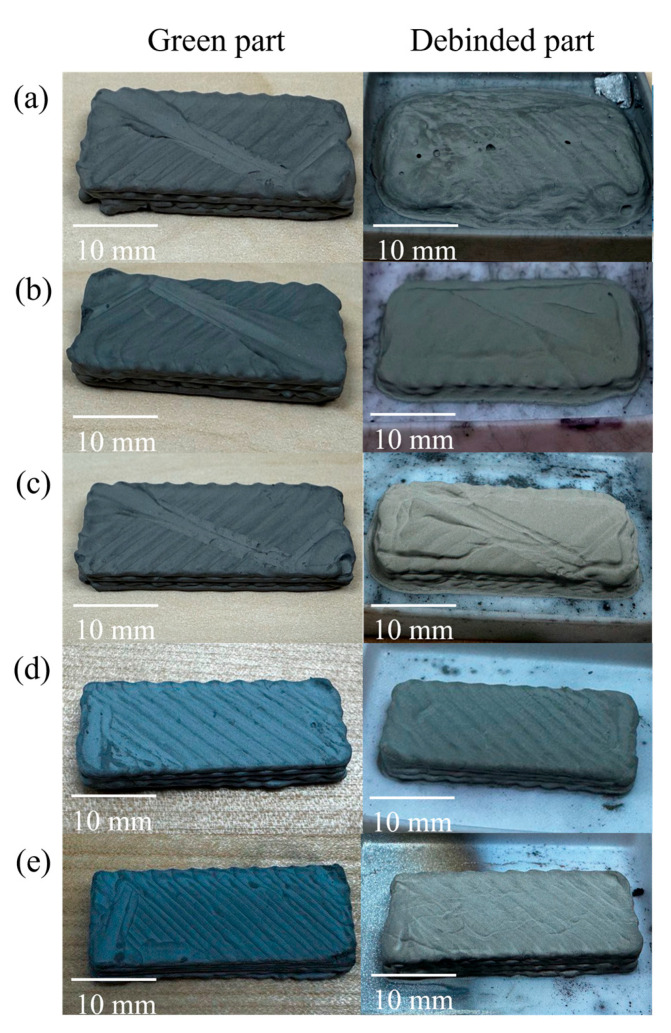
Images of green and debinded printed parts for feedstocks with metal contents: (**a**) 93.0 wt.%, (**b**) 94.0 wt.%, (**c**) 94.5 wt.%, (**d**) 95.0 wt.%, and (**e**) 95.5 wt.%. (**Left column**): green state, (**right column**): debinded state. Scale bars: 10 mm.

As depicted in Figure 10, samples with higher metal contents (95.0 wt.% and 95.5 wt.%) maintained their structural stability after thermal debinding. In contrast, samples with lower metal contents (93.0 wt.%, 94.0 wt.%, and 94.5 wt.%) exhibited deformation and loss of shape integrity. This behavior is primarily attributed to the insufficient metal particle framework in lower-metal-content feedstocks, which fails to provide the necessary mechanical support once the binder is removed. In addition to structural packing, inter-particle friction plays a crucial role during thermal debinding, especially in highly loaded systems. As described in the MIM literature [37], higher metal content enhances particle-to-particle contact, increasing frictional resistance to distortion during binder burnout. In lower-loaded systems, particles are more widely spaced and rely heavily on the binder, leading to collapse or warping once the binder decomposes. This effect complements observations by Mao et al. [36], who reported that increasing solid loading improved green part stability during binder jetting debinding.

**Figure 11:**
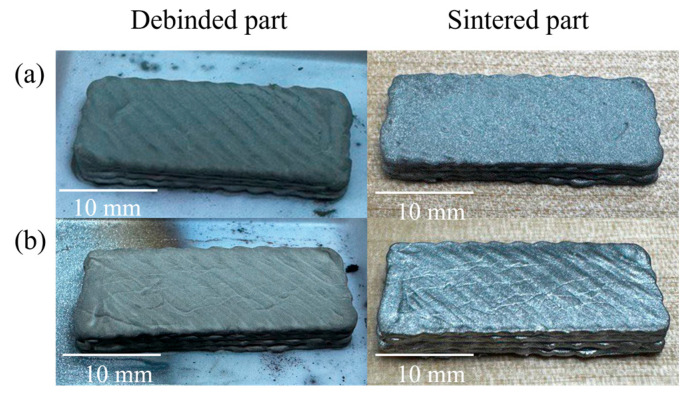
Image of debinded (**left**) and sintered (**right**) parts for feedstocks with metal contents: (**a**) 95.0 wt.% (**top row**) and (**b**) 95.5 wt.% (**bottom row**). Scale bars: 10 mm.

Figure 11 shows that the sintered parts produced from 95.0 wt.% and 95.5 wt.% metal-loaded feedstocks successfully retained their dimensional integrity and surface definition after sintering at 1340 °C. The absence of warping, cracking, or structural collapse indicates effective consolidation and thermal stability throughout the sintering process. In addition, no indicators of hydrogen embrittlement were observed, so the mechanical behavior is going to be consistent with porosity-limited, as-sintered 17-4PH rather than hydrogen-assisted fracture [60].

This outcome reflects the strong foundation provided by the high-quality green parts, particularly those with increased powder content, which exhibited higher green density and stiffness. The dense particle network in these feedstocks enhances mechanical rigidity and inter-particle friction, both of which are critical in supporting the part during debinding and early sintering stages when the structure is most vulnerable [37].

Superior shape retention is consistent with prior studies such as Wagner et al. [61], who reported that feedstocks with high solid loading maintained excellent dimensional stability when sintered near 1350 °C. Similar trends have been observed in extrusion-based AM of 316L stainless steel, where optimized powder-binder ratios contributed to robust sintered geometries [62].

### 3.5. Sintered Part Properties

**Figure 12:**
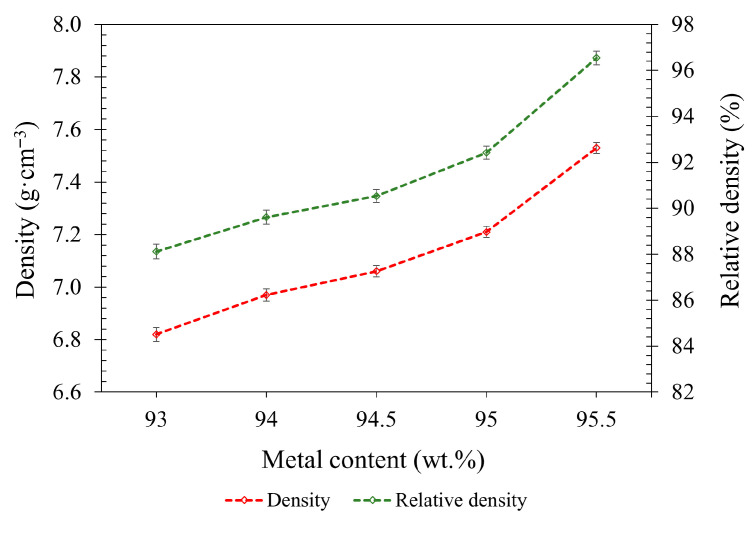
Density and RD values for as-sintered parts.

Figure 12 shows that increasing the metal content from 95.0 wt.% to 95.5 wt.% significantly enhances both as-sintered density and RD, rising from 7.2 to 7.5 g·cm^−3^ and 92.4% to 96.5%, respectively. Despite the small increase in metal loading, the densification improvement is substantial, possibly indicating that the system is sensitive to changes near the critical powder loading threshold [51]. This trend aligns with the behavior of the green parts, where the relative green density increased from 56.2% to 59.1%. Higher powder content leads to better particle packing and reduced binder volume, improving both the green structure and sintering response [51,63].

**Figure 13:**
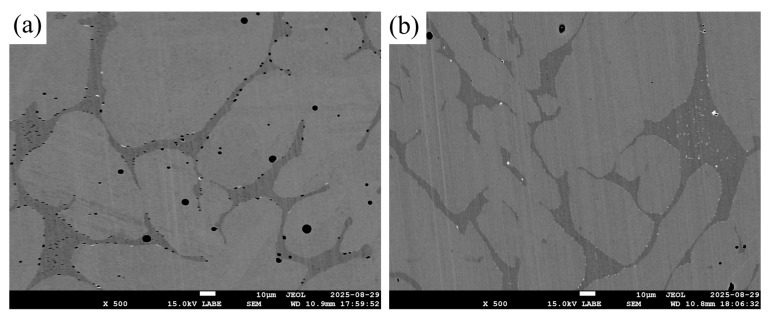
SEM-BSE micrographs (unetched) of as-sintered 17-4PH made by metal FDM: (**a**) F4 (95.0 wt.%), (**b**) F5 (95.5 wt.%).

Figure 13 presents SEM-BSE micrographs of the as-sintered microstructure for the two feedstocks. For F4 (95.0 wt.% metal, 92.4% RD) (Figure 13a) the section shows a higher fraction of elongated, layer-aligned voids with smaller necks, consistent with incomplete closure of printing-induced inter-layer gaps at the lower solid loading [64]. In contrast, F5 (95.5 wt.% metal, 96.5% RD) (Figure 13b), exhibits predominantly isolated, near-equiaxed pores with well-developed inter-particle necks, indicative of advanced densification [65]. Intergranular or quasi-cleavage features characteristic of hydrogen-assisted damage were not observed, the microstructural differences are governed by porosity and neck growth [66].

**Figure 14:**
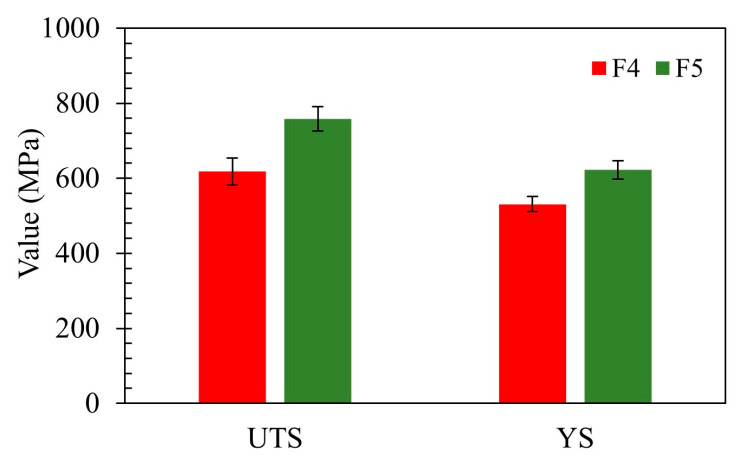
Tensile properties (UTS and YS) of as-sintered 17-4PH samples from feedstocks F4 (95.0 wt.%) and F5 (95.5 wt.%). Error bars indicate standard deviation based on three specimens per condition.

Figure 14 illustrates the comparative tensile properties, ultimate tensile strength (UTS) and 0.2% offset yield strength (YS), for machine-sintered tensile specimens produced from feedstocks with 95.0 wt.% (F4) and 95.5 wt.% (F5) metal powder. The detailed values, including elongation at break, are presented in Table 5. The mechanical strength of sintered FDM fabricated specimen as shown in Table 5 are consistent with the as-sintered ranges reported for FDM or bound-powder-extrusion 17-4PH in prior studies [67]. It is approximately 45% lower than classical cast and wrought bar (UTS ≈ 1.38–1.40 GPa, YS ≈ 1.25–1.28 GPa, elongation ≈ 11–14% [68]) and 14% lower than laser powder bed fusion (LPBF) fabricated specimen in as-built conditions (UTS ≈ 0.89–0.92 GPa, YS ≈ 0.86 GPa, elongation ≈ 20% [69]). However, previous works [26,70] show the strength of sintered FDM specimen enhances post precipitation aging and matches with that of post processed cast and wrought and LPBF conditions. AM techniques enable fabrication of complex near net shape geometry components as compared to classical techniques. Moreover, FDM offers practical benefits over other AM processes such as LPBF and electron beam melting (EBM) with its simpler workflow, lower capital and operational cost [71].

All specimens displayed characteristic elastic–plastic deformation followed by fracture. However, increasing the powder content resulted in a significant improvement in mechanical performance. The F4 samples exhibited lower strength, with an average UTS of 618 MPa, YS of 531 MPa, and elongation at break of 1.5%. In contrast, the F5 samples demonstrated enhanced performance, achieving a UTS of 758 MPa, YS of 622 MPa, and elongation of 5.2%.

These enhancements are attributed to improved particle packing and reduced binder content in the 95.5 wt.% feedstock. This densification facilitates more effective diffusion bonding during sintering and minimizes residual porosity, resulting in a stronger, more cohesive metallic network [24,51,72].

**Figure 15:**
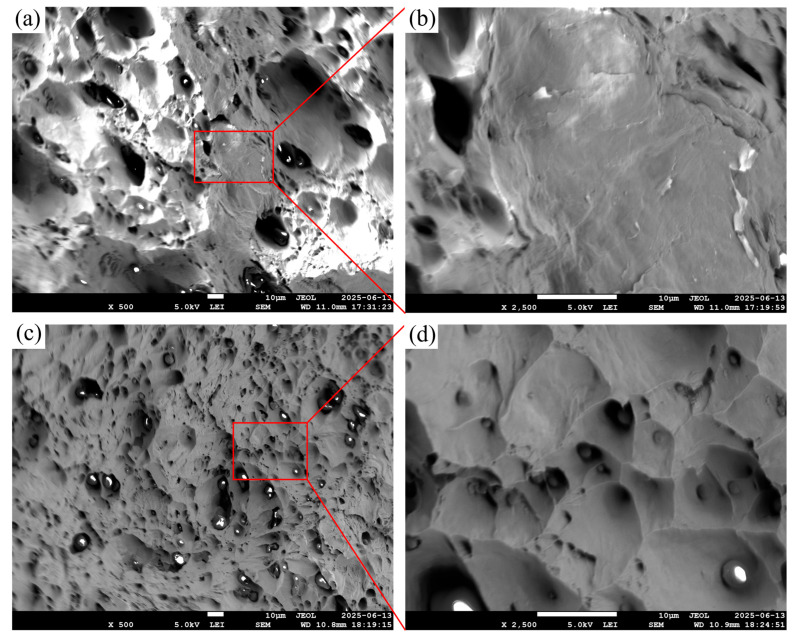
SEM images of fracture surfaces: (**a**,**b**) F4 (95.0 wt.%) showing brittle features, (**c**,**d**) F5 (95.5 wt.%) showing ductile fracture with dimples.

Fracture surface analysis using SEM (Figure 15) corroborates these mechanical trends. The F4 fracture surfaces (Figure 15a,b) reveal a relatively flat morphology with few plastic deformation features, indicative of brittle fracture behavior [73,74]. In contrast, the F5 samples (Figure 15c,d) show a highly dimpled and irregular surface, characteristic of ductile fracture, with extensive micro void coalescence [75]. The increase in ductile features aligns with the higher elongation values observed in tensile testing. In neither case do we observe intergranular or quasi-cleavage features characteristic of hydrogen embrittlement [76].

The transition from brittle to ductile fracture with increased metal powder loading reflects improved matrix continuity and reduced stress concentrations due to lower porosity. These microstructural refinements enable stable plastic flow and delayed crack propagation, ultimately leading to simultaneous improvements in both strength and ductility [77,78].

### 3.6. Tribocorrosion Performance

**Figure 16:**
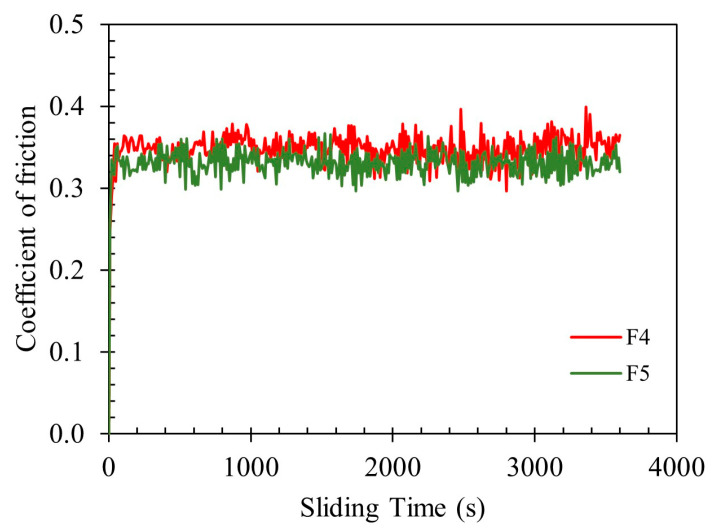
Coefficient of friction vs. time of as-sintered 17-4PH samples from feedstocks F4 (95.0 wt.%) and F5 (95.5 wt.%), during tribocorrosion testing.

Figure 16 presents the coefficient of friction (CoF) as a function of time for polished-sintered samples F4 (95.0 wt.%) and F5 (95.5 wt.%) during the tribocorrosion test. Both samples exhibit a typical running-in period followed by a steady-state friction regime. Notably, sample F5 demonstrated consistently lower average CoF compared to F4 throughout the test duration, indicating a more stable and lubricated contact interface. Consistent with these observations, the wear coefficient was lower for F5 (2.1 × 10^−5^ mm^3^· (N·m)^−1^) than for F4 (3.4 × 10^−5^ mm^3^·(N·m)^−1^). The reduced friction and wear in F5 can be attributed to its higher RD, which likely enhanced the load-bearing capacity and minimized surface damage during sliding. These results suggest that increasing the density of sintered parts improves tribocorrosion resistance by simultaneously reducing both mechanical wear and frictional losses [79].

## 4. Conclusions

This study successfully formulated and characterized highly filled 17-4PH feedstocks using a sustainable, low-molecular-weight binder system composed of PW and SA, designed for metal FDM. The major conclusions from this study are as follows:

- Rheology showed shear-thinning for all formulations, with viscosity increasing sharply with solid loading.
- Increasing metallic loading by 2.5 wt.% improved flexural modulus by ≈33%, green density by ≈12%, whereas flexural strength reduced slightly by ≈6%.
- Only the 95.0 and 95.5 wt.% compositions maintained dimensional stability through debinding and sintering, indicating adequate green robustness and packing.
- Increasing the metal content from 95.0 to 95.5 wt.% improved as-sintered performance markedly, raising relative density by 4.1 percentage points and enhancing tensile properties (UTS +≈23%, YS +≈17%, and elongation +3.7 percentage points, ≈3.5×).
- Tribocorrosion performance improved with higher solid loading. Increasing the metal content from 95.0 to 95.5 wt.% reduced the wear coefficient by ≈38%, accompanied by a concomitant decrease in the coefficient of friction.

In conclusion, for low-strength eco-friendly polymers, a viable strategy is to overload the binder with metallic powder to compensate for the polymer’s mechanical weakness by leveraging friction forces between densely packed metal particles. However, this friction-based structural stability is ultimately constrained by the printer’s ability to process the resulting high-viscosity feedstock, which defines the upper limit of viable powder loading.

## Figures and Tables

**Table 1:** Chemical composition of 17-4PH for gas atomized powder alloys (wt.%).

Fe	Cr	Ni	Mn	Si	C	Cu	Nb + Ta
Bal.	16–17	4–5	≤1.0	≤1.0	≤0.07	4	0.3

**Table 2:** Formulations of the investigated feedstocks (wt.%).

Sample	Metal Powder (wt.%)	PW (wt.%)	SA (wt.%)
F1	93.0	5.0	2.0
F2	94.0	4.3	1.7
F3	94.5	3.9	1.6
F4	95.0	3.6	1.4
F5	95.5	3.2	1.3
F6	96.0	2.9	1.1

**Table 3:** DOE for printing parameters.

Temperature (°C)	Printing Speed (mm·s^−1^)	Flow Rate (mm^3^·s^−1^)
80	10	5
80	7	10
80	5	15
90	5	15
90	7	10
90	10	5
95	5	15
95	7	5
95	10	10

**Table 4:** Optimum printing parameters for the feedstocks.

Sample	Nozzle Size (mm)	Layer Height (mm)	Temperature (°C)	Printing Speed (mm·s^−1^)	Flow Rate (mm^3^·s^−1^)
F1	1.2	1	80	10	5
F2	1.2	1	90	10	5
F3	1.2	1	90	7	10
F4	1.2	1	90	7	10
F5	1.2	1	95	5	15

**Table 5:** Tensile properties of as-sintered 17-4PH specimens.

Sample	UTS (MPa)	YS (MPa)	Elongation at Break (%)
F4	618 ± 36	531 ± 25	1.5 ± 0.08
F5	758 ± 32	622 ± 25	5.2 ± 0.21

## Data Availability

The data presented in this study is available on request from the corresponding author. The data is not publicly available due to institutional and industrial confidentiality agreements.
